# The association between men working abroad and the health of their pregnant wives and newborns in Tajikistan. A secondary analysis of demographic and health survey data

**DOI:** 10.1016/j.jmh.2026.100450

**Published:** 2026-10-16

**Authors:** J․Mirjam Oudijk, Jelle Stekelenburg

**Affiliations:** a MSc Public Health student, London School of Hygiene and Tropical Medicine, United Kingdom; b Assistance International, Dushanbe, Tajikistan; c University of Groningen, University Medical Center Groningen, Department of Health Sciences, Global Health Unit, Groningen, the Netherlands; d Frisius MC, Department of Obstetrics & Gynaecology, Leeuwarden, The Netherlands

**Keywords:** Migrants, Wives, Newborns, DHS, Antenatal care, Birthweight

## Abstract

**Background:**

High rates of international labour migration have consequences on the health of wives and children left behind by their husbands or fathers, which is sparsely investigated. This study evaluates the impact of migrant husbands on the left-behind wives’ antenatal care and the health of their newborns, comparing wives of migrated and not-migrated husbands from Tajikistan.

**Methods:**

Data from the Tajikistan 2017 Demographic and Health Survey was analysed. 3057 women who had a child under the age of three years and whose husband’s migration status was known were included in this analysis. Descriptive and multivariable logistic regression analysis was performed. Starting antenatal care (ANC) in the first trimester and low birthweight (LBW) were the main outcomes investigated.

**Results:**

In the last three years, 42.5% of husbands worked abroad for at least three months. In addition, 68.3% of women started ANC in the first trimester, 6.5% of newborns had LBW (<2500 gram). Wives of migrants had 20% lower odds of timely ANC and 37% lower odds to have a child with LBW, in comparison with wives of non-migrants. Parental higher education increased the odds of timely ANC. Higher paternal education level and mothers involved in decision-making about health reduced the odds of LBW.

**Conclusion:**

Wives of migrants started ANC later, but their newborns had higher birthweights compared to wives of non-migrants. Early ANC and birthweight improved with father’s education level and mother’s autonomy. Future interventions should address family’s awareness of the need for ANC especially when fathers plan to migrate.

## Highlights

• Men working abroad impacts the health of their wives and newborns.
• Women in Tajikistan whose husbands work abroad start antenatal care later.
• Newborns in Tajikistan had higher birthweights when the father worked abroad.
• Mothers’ involvement in decision-making also influences the health of their newborns.

## 1 Introduction

Many Tajik men leave for Russia to ensure a better future for their families. Many of them leave a pregnant wife behind. The impact of such migration on their pregnant wives’ health, healthcare utilisation and pregnancy outcomes is unknown.

Tajikistan is a Central Asian country that belonged to the Soviet Union until independence in 1991, but still highly depends on Russia. A large proportion of Tajik men, and some women, work abroad as migrants, mostly in Russia, up to almost half of the male working population (World Bank Group, 2023). Typically, migrants go to Russia from spring till autumn, but a substantial amount stays in Russia for years or never returns home (IOM Dushanbe, 2009).

Research from within Tajikistan on health-related behaviours and outcomes for wives and children left behind has been scarce, mostly not peer-reviewed and showing mixed results: more doctor’s visits, attributed to receiving remittances, but also more sexually transmitted infections (Miskinzod, 2015); delays in seeking obstetric care (Nabieva and Souares, 2019); more depression in left-behind wives, but more post-traumatic stress disorders in women without a migrated husband (Pirova et al., 2018).

Findings from other countries on the effect of male migration also showed mixed results. Studies looking at women’s health status reported reduced mortality in women whose husbands were successful migrant workers (Agadjanian et al., 2021) while other studies described more mental health problems (Aryal et al., 2019; Lu, 2012) or worse general physical health, less underweight but more overweight in such women (Lei and Desai, 2021; Sznajder et al., 2021; Ullmann, 2012). An increase in mother-and-child-health service utilisation was seen but skilled birth attendance or facility birth did not always increase (Atake, 2018; Khan et al., 2023). One study described reduced awareness and use of family planning and reproductive health-services (Shattuck et al., 2019). Another study reported increased gestational weight gain and decreased incidence of preterm births (Jayatissa et al., 2022). In addition, increased HIV awareness and communication was seen in one study but condom use did not increase (Agadjanian and Markosyan, 2017).

In regard to children, findings from Tajikistan showed less malnutrition in children from households and communities where more men migrated (Azzarri and Zezza, 2011). Children had increased access to healthcare and better nutrition, but they also had more psychosocial problems (UNICEF Tajikistan, 2011). Research from other countries on the impact of parental migration on children mostly describe short term negative effects on the child’s psychosocial health. This varied with the gender of the child (more behavioural problems in boys and emotional problems in girls) and of the migrating parent (more negative effects when the mother migrates) (Antia et al., 2020; Fellmeth et al., 2018; Graham and Jordan, 2011; Mazzucato et al., 2015; Penboon et al., 2019; Vanore et al., 2015; Wickramage et al., 2015). However, negative effects on mental health were not seen long term (Norori et al., 2025). Concerning physical health, measured by children’s growth, mixed results were reported; positive effects (Kyrgyzstan), negative effects (Sri Lanka, India, Peru and Vietnam) or no effect (Bangladesh) (Islam et al., 2019; Lu, 2015; Račaitė et al., 2021; Viet Nguyen, 2016; Wickramage et al., 2015; Wigle et al., 2020).

Countries that observed a positive relationship between migrant men and health outcomes of their family attributed this to the remittances and increased use of private healthcare (Atake, 2018; Jayatissa et al., 2022). While Tajikistan highly depends on remittances, it has a very limited private healthcare sector and not every woman receives remittances, so findings from other countries may not be applicable to Tajikistan (Asian Development Bank, 2023; IOM Dushanbe, 2009; Khodjamurodov et al., 2016; Migrants and Refugees Section, 2020). Characteristics of the health system in Tajikistan are that it focuses on in-patient care more than primary care and suffers from low quality of care, due to a lack of evidence-based guidelines and equipment, insufficient training and other factors (Khodjamurodov et al., 2016). Maternal and infant mortality, though declining, remain one of the highest of the former Soviet Union countries (UN Inter-agency Group for Child Mortality, n.d.; UNICEF, n.d.). Healthcare is mostly funded by out-of-pocket payments, which creates a heavy burden for many families (Khodjamurodov et al., 2016).

It is not clear how male migration from Tajikistan affects their pregnant wives and newborns. This study therefore investigates how absence of husbands impacts the health-related behaviours of pregnant women and the health outcomes of their newborns left behind in Tajikistan. It specifically explores whether there is an association between husbands being away as migrants (compared to husbands not leaving the country) and maternal health factors (antenatal care (ANC) visits, facility births) or child health factors (birthweight, size of child at birth, breastfeeding status, mortality and having had a postnatal check).

## 2 Methods

### 2.1 Study design and population

This is a cross-sectional study with a secondary data analysis from the Tajikistan 2017 Demographic and Health Survey (DHS) (SA, MOHSP and ICF, 2018), a nationally representative household survey. DHS data is made available on request for academic purposes. The Tajikistan 2017 DHS added this country-specific question: “Did your husband work abroad for at least three months in the last three years?” which forms the exposure variable for this study. Women who reported their husbands worked abroad as migrants were compared to those whose husbands did not leave the country for work. The analysis was restricted to mothers who gave birth to a child in the last three years. Maternal outcome variables used include starting ANC in the first trimester, number of ANC visits, birth in a health-facility. Newborn outcome variables used include mortality, birthweight, size of child at birth, still breastfeeding at the time of interview, having had a postnatal check within two months of birth.

Out of 10,718 interviewed women, 3166 women had a child born in the last 36 months. Only data from the lastborn was used. Women without a partner (75) and women whose lastborn were twins or triplets (34) were excluded from the study. In total, 3057 singleton births were included in the study.

### 2.2 Variables

290 women had missing data for timely ANC (10), number of ANC visits (9), place of delivery (8), birthweight (237), size of child at birth (122), postnatal check (14) and husband’s education level (4). These were included in the descriptive analysis but excluded from regression analysis. The number of women with missing data was (9.5%). Missing data for birthweight (one of the main outcomes) was examined for association with migration status and covariables. The other main outcome, timely ANC, was only missing for ten women.

Timely ANC was categorised as ANC started on time (in first trimester) or late (fourth month or later). Number of ANC visits were categorised as: no or <4 visits, 4–7 visits and 8 visits or more, as in the past the World Health Organization (WHO) advised a minimum of 4 visits, but currently advises a minimum of 8 visits (WHO, 2016). Place of delivery was categorised as facility-birth or outside of facility.

Timely ANC was chosen as the main maternal outcome for subsequent multivariable modelling, determined after the descriptive analysis, based on the strength of the crude association (as pre-specified per protocol). The other outcomes are reported as additional outcomes.

The variable mortality refers to whether the child was alive at the time of the interview. Because of small numbers, no distinction was made whether this was in the neonatal period or later.

DHS collected both the ‘birthweight’ and ‘size of child at birth’. The variable ‘birthweight’ was missing for 237 newborns (190 were not weighed at birth, 47 unknown weight). For size of child at birth, mothers were asked whether the child was either very small, small, average, large or very large at birth. This question was answered as ‘don’t know’ by 122 mothers. Size of child was categorised: ‘small’ (those very small and smaller than average) or ‘average or large’ (average, larger than average, or very large). Birthweight was categorised as <2500 *g* (LBW) and ≥2500 *g* (normal birthweight). Birthweight was selected as main outcome to explore in multivariable analysis because of its stronger association (crude odd ratio) with migration status in the unadjusted analysis (as pre-specified per protocol). The other outcomes are reported as additional outcomes.

DHS 2017 did not collect data on the duration of breastfeeding. To assess any difference in breastfeeding practices, the variable whether the mother is still breastfeeding or not at the time of the interview was used.

Postnatal check refers to the child having had a postnatal check within the first two months after birth.

### 2.3 Description and categorisation of possible confounders

The variables examined as possible confounders were socioeconomic status (wealth quintile), maternal age, maternal education level, husband’s education level, rural or urban setting, region, age of last child, gender of the child, whether the child is the mother’s firstborn and mother’s decision-making position in the family.

Mother’s age was grouped in 5-year bands in the DHS. Because of small numbers in the youngest and oldest age groups, groups were combined to a total of 3 groups: 18–24, 25–29, 30 years and older. Mother’s education level was categorised into three groups (up to 9th grade, finished 10th or 11th grade, and having further education), reflecting the most common durations of education in Tajikistan. Education in years was not available for the husbands, but only whether they have no education, primary, secondary or higher education. This was categorised into two groups: having received higher education or not.

Region refers to the five administrative regions of Tajikistan (the capital and four provinces), as used in DHS: Dushanbe, Districts of Republican Subordination (DRS), Sughd, Khatlon, Gorno-Badakhshan Autonomous Region (GBAO). The northern province of Sughd was chosen as baseline.

Mother’s decision-making position in the family refers to being involved in decisions related to health. This was categorised into two groups: involved in decision-making (alone or together with her husband) or not involved (the husband or someone else makes decisions). As only 228 (7.5%) women were making decisions about health alone, this was not included as a separate group. Where the DHS data said ‘other’, this was interpreted as the mother not being involved in the decision-making about health.

### 2.4 Statistical analysis

The statistical analysis included descriptive statistics for the distribution of study participants and all variables included in the study. Chi square and Wald tests were used to explore the characteristics of the study population.

Possible confounders were explored for their association with migrant work and the maternal and newborn outcome variables, using logistic regression and Wald tests.

Multiple logistic regression analysis was performed to investigate the association between the primary exposure (husband working abroad in the past three years compared to those not working abroad) and the maternal (timely ANC) and newborn (birthweight) outcome variables adjusting for all possible confounders, obtaining adjusted odds ratios and confidence intervals with Wald tests. Possible interaction was assessed with the adjusted Wald test.

All variables were included in the final logistic regression model. All variables identified as confounders in the univariate analysis were added to the final multivariable model. Except for where there might be collinearity between variables, these were removed from the regression model.

The statistical analysis was performed with Stata 16.1.

All analysis took weightings for sampling and survey design into account, using Stata’s ‘survey’ function and the weighting factors that were already included in the DHS data, based on region, urban vs rural setting and clusters (SA, MOHSP and ICF, 2018). Numbers presented in this methods section are still unweighted numbers.

### 2.5 Ethical considerations

The DHS was conducted with prior ethical approval from the appropriate boards in the United States and Tajikistan. All women had given informed consent prior to the DHS interview. Ethical permission from the London School of Hygiene and Tropical Medicine was obtained for the secondary data analysis for the MSc project.

## 3 Results

### 3.1 Socio-demographic characteristics

A weighted number of 3220 women were included in the analysis, based on the actual sample of 3057 women with singleton births (Table 1). Numbers reported from here onwards are weighted numbers, unless otherwise stated. Most women were between 18 and 24 years old (1208 women) or between 25 and 29 years old (1106 women). The median age of their youngest child was 15 months (interquartile range 8–24 months) and there were slightly more boys than girls (51.0 vs 49.0%). For 25% of women this was their first child. Only 14.8% of women and 28.1% of husbands had received higher education. About 80% lived in rural areas. Two-thirds of women were not involved in decision-making about their health. Most women came from Sughd, Khatlon or DRS region, 6.0% lived in Dushanbe and 1.7% in sparsely populated GBAO. Dushanbe was considered 100% urban.

**Table 1:** Characteristics of study population and association with husband’s migration.

Variable	Total number (%)	Non-migrant husband (%)	Migrant husband (%)	p-value^b^
Total number of women	3220 (100)	1853 (57.5)	1367 (42.5)	-
Median age of last child in months (interquartile range)	15 (8–24)	15 (7–23)	16 (8–24)	0.050
Gender of last child				0.063
Male	1641 (51.0)	977 (52.7)	664 (48.6)	
Female	1579 (49.0)	876 (47.3)	703 (51.4)	
Maternal age				0.347
18–24 year	1208 (37.5)	678 (36.6)	529 (38.7)	
25–29 year	1106 (34.4)	644 (34.8)	462 (33.8)	
30 year and older	906 (28.1)	530 (28.6)	376 (27.5)	
Maternal education				0.061
None to grade 9	1406 (43.7)	780 (42.1)	626 (45.8)	
Grade 10–11	1337 (41.5)	780 (42.1)	557 (40.8)	
Further education	477 (14.8)	292 (15.8)	184 (13.5)	
Paternal education^a^				<0.001
Finished grade 11	2314 (72.0)	1204 (65.0)	1111 (81.4)	
Further education	902 (28.1)	649 (35.0)	253 (18.6)	
Rural or urban setting				<0.001
Urban	637 (19.8)	456 (24.6)	181 (13.2)	
Rural	2583 (80.2)	1397 (75.4)	1187 (86.8)	
Region				0.320
Sughd	948 (29.4)	492 (26.5)	456 (33.4)	
Dushanbe	194 (6.0)	153 (8.3)	40 (3.0)	
Khatlon	1249 (38.8)	742 (40.1)	507 (37.1)	
DRS	776 (24.1)	427 (23.1)	348 (25.5)	
GBAO	54 (1.7)	38 (2.1)	16 (1.1)	
Wealth quintile				0.027
Poorest	599 (18.6)	357 (19.3)	242 (17.7)	
Poorer	656 (20.4)	350 (18.9)	306 (22.4)	
Middle	730 (22.7)	383 (20.7)	347 (25.4)	
Richer	711 (22.1)	387 (20.9)	324 (23.7)	
Richest	523 (16.3)	375 (20.3)	148 (10.8)	
Firstborn child				0.325
Yes	788 (24.5)	440 (23.7)	349 (25.5)	
No	2432 (75.5)	1413 (76.3)	1019 (74.5)	
Mother’s decision-making about health				0.684
Involved	1087 (33.8)	618 (33.4)	469 (34.3)	
Not-involved	2133 (66.2)	1234 (66.6)	898 (65.7)	

Numbers are weighted counts. Numbers may not add up to the total because of weightings and rounding.

a n=3217;

b Wald test p-value for association with migration for each variable.

A total of 1367 husbands (42.5%) worked abroad for at least three months in the last three years (Table 1). Migrant work was more prevalent among families from rural areas (86.8% of migrants and 75.4% of non-migrants were from rural areas, p < 0.001), men without further education (81.4% of migrants and 65.0% of non-migrants were without further education, p < 0.001) and those not from the highest or lowest wealth quintile. In unadjusted analyses with covariates, migration was not associated with maternal age, having their first child and mother’s decision-making position in the family. The percentage of migrated husbands varied hugely per region, from 21% in Dushanbe to 48% in Sughd (not shown in table).

### 3.2 Missing variables

Birthweight was missing for 7.8% of women and this was weakly associated with husband migration status (chi square p = 0.09) and more strongly associated with maternal age, maternal education level, urban setting, region, wealth quintile, firstborn child and mothers’ involvement in decision-making about health (for each of these variables chi square p < 0.05).

### 3.3 Univariate analysis

All eight outcome variables were included in the univariate analysis, see Table 2. 68.3% of women started ANC in the first trimester.

**Table 2:** Maternal and newborn health outcomes with husband migration status and crude odds ratios, comparing wives of migrant to non-migrant husbands.

Outcome variable	Women (%)	Non-migrant husband (%)	Migrant husband (%)	OR^a^	95%-CI	p-value^b^
Timely ANC^c^ (n = 3210)						
Yes	2193 (68.3)	1302 (70.5)	891 (65.4)	0.79	0.65–0.97	0.023
No	1017 (31.7)	545 (29.5)	471 (34.6)	1.00		
ANC frequency (n = 3212)						
0–3 visits	1128 (35.1)	653 (35.3)	475 (34.8)	0.99^d^	0.78–1.25	0.909^e^
4–7 visits	1615 (50.3)	930 (50.3)	686 (50.3)	1.00		
8 or more visits	470 (14.6)	266 (14.4)	203 (14.9)	1.03^d^	0.79–1.34	0.806^e^
Facility-birth (n = 3212)						
Yes	2855 (88.9)	1630 (88.3)	1224 (89.6)	1.14	0.85–1.53	0.391
No	357 (11.1)	215 (11.7)	142 (10.4)	1.00		
Child died (n = 3220)						
Yes	54 (1.7)	31 (1.7)	23 (1.7)	1.03	0.55–1.94	0.925
No	3166 (98.3)	1822 (98.3)	1344 (98.3)	1.00		
Low birthweight (<2500 g) (n = 2969)						
Yes	194 (6.5)	129 (7.6)	65 (5.1)	0.66	0.46–0.93	0.020
No	2775 (93.5)	1563 (92.4)	1212 (94.9)	1.00		
Size of child at birth (n = 3090)						
Small	448 (14.5)	281 (15.9)	167 (12.6)	0.76	0.59–0.99	0.044
Average or large	2642 (85.5)	1487 (84.1)	1155 (87.4)	1.00		
Still breastfeeding (n = 3220)						
Yes	1854 (57.6)	1098 (59.3)	756 (55.3)	0.85	0.71–1.02	0.074
No	1366 (42.4)	755 (40.7)	611 (44.7)	1.00		
Postnatal check <2months (n = 3209)						
Yes	1990 (62.0)	1146 (62.1)	844 (61.9)	0.99	0.81–1.22	0.930
No	1219 (38.0)	699 (37.9)	520 (38.1)	1.00		

Numbers are weighted counts. Numbers may not add up to the total because of weightings and rounding.

a ORs for association with migration, calculated with logistic regression.

b P-values from Wald test for association with migration.

c Timely ANC: started in the first trimester.

d OR for ANC frequency calculated with multinomial logistic regression.

e Overall p-value could not be obtained due to restraints with using survey settings in Stata.

Wives of migrants were 21% less likely to start ANC in the first trimester compared to wives of non-migrant men (unadjusted OR 0.79, 95%-CI 0.65–0.97, p = 0.02). There was no evidence for an association of migration status with ANC frequency and facility births (see Table 2).

Newborns of migrant men were less likely to have LBW (OR 0.66, 95%-CI 0.46–0.93, p = 0.02) or a ‘small or very small’ size at birth (OR 0.76, 95%-CI 0.59–0.99, p = 0.04) compared to newborns of non-migrants. There was no effect on current breastfeeding status when corrected for child age (bivariable logistic regression OR 0.91, 95%-CI 0.73–1.13, p = 0.39, not shown in table). There was no difference in mortality (OR 1.03) or having a postnatal check (OR 0.99).

Based on these findings, ‘timely ANC’ as maternal factor and ‘birthweight’ as newborn factor were selected to be included in further multivariable analysis of the association with migrant status.

### 3.4 Bivariate analysis

Adjusting for each variable in turn (Table 3), each potential confounder had <10% effect on the OR for the association between migration and starting ANC on time. Region had a small negative confounding effect (adjusted OR 0.75, 95%-CI 0.62–0.93, p = 0.007) while paternal education had a small positive effect and made the association no longer significant (adjusted OR 0.85, 95%-CI 0.70–1.04, p = 0.11). In other words, region had led to an underestimation of the association between migration and timely ANC, and paternal education had led to an overestimation of this association. For LBW, findings were similarly small. The largest confounding effect was found for paternal education, which had a negative (underestimating) confounding effect (adjusted OR 0.62, 95%-CI 0.44–0.88, p = 0.008).

**Table 3:** Crude and adjusted associations of husband’s migration with timely ANC and with LBW, comparing migrant with non-migrant husbands, adjusted for each variable in turn.

Variable	OR^a^ timely ANC^b^ (n = 3210)	95%-CI	p-value^c^	OR^a^ LBW (n = 2969)	95%-CI	p-value^c^
Unadjusted effect of migration status	0.79	0.65–0.97	0.023	0.66	0.46–0.93	0.020
*Effect of migration adjusted for:*						
Age of child^d^	0.80	0.65–0.97	0.025	0.65	0.46–0.93	0.018
Gender	0.79	0.65–0.97	0.022	0.65	0.46–0.93	0.017
Maternal age	0.78	0.64–0.96	0.018	0.65	0.46–0.93	0.017
Maternal education	0.81	0.66–0.99	0.042	0.66	0.46–0.93	0.020
Paternal education^e^	0.85	0.70–1.04	0.109	0.62	0.44–0.88	0.008
Urban vs rural	0.83	0.68–1.02	0.078	0.65	0.45–0.93	0.018
Region	0.75	0.62–0.93	0.007	0.67	0.47–0.95	0.026
Wealth quintile	0.80	0.65–0.98	0.029	0.66	0.46–0.95	0.025
Firstborn	0.78	0.64–0.96	0.017	0.65	0.46–0.93	0.017
Mother’s decision-making^f^	0.79	0.65–0.96	0.020	0.66	0.46–0.93	0.019

Based on weighted data (adjusted for clustering and stratification).

a ORs calculated with logistic regression.

b timely ANC: ANC started in the first trimester.

c p-values from Wald test for adjusted relationship between migration and outcome (timely ANC or birthweight).

d age is a continuous variable (months).

e n = 3207 and 2967 respectively.

f mother’s decision-making: involved in decisions about health.

### 3.5 Multivariable logistic regression

In the multivariable model which included all potential confounders (Table 4), there is still strong evidence for an association between migration and timely ANC (adjusted OR 0.80 95%-CI 0.66–0.98, p = 0.03), which does not differ much from the crude OR of 0.79. In other words, women whose husbands had worked abroad had 20% lower odds to start ANC in the first trimester than women whose husbands had not worked abroad, when adjusted for age of the child, gender, maternal age, maternal education level, paternal education level, urban setting, region, firstborn child and mothers’ involvement in decision-making about health.

**Table 4:** Multivariable analysis, adjusted estimates of the odds ratios for ‘timely ANC’ and ‘low birthweight’ estimated by logistic regression.

	**OR timely ANC**^a^**(n** **=** **3207)**	**95%-CI**	**p-value**^b^	**OR Birthweight <2500 *g* (n** **=** **2967)**	**95%-CI**	**p-value**^b^
**Effect of migration**	0.80	0.66–0.98	0.032	0.63	0.44–0.89	0.009
***Adjusted OR for effect of other variables in model:***						
**Age of child**^c^	1.00	0.99–1.01	0.444	1.01	1.00–1.03	0.152
**Gender**						
Male	1.00			1.00		
Female	1.03	0.85–1.24	0.747	1.18	0.85–1.64	0.316
**Maternal age**						
18–24 year	1.00			1.00		
25–29 year	1.01	0.78–1.31	0.944	0.86	0.55–1.36	0.525
30 year and older	0.67	0.52–0.86	0.002	0.88	0.48–1.60	0.677
**Maternal education**						
None to grade 9	1.00			1.00		
Grade 10–11	1.18	0.94–1.49	0.153	1.27	0.85–1.90	0.247
Further education	1.77	1.32–2.39	<0.001	1.17	0.59–2.30	0.653
**Paternal education**						
None to grade 11	1.00			1.00		
Further education	1.36	1.07–1.73	0.013	0.67	0.45–1.00	0.051
**Urban vs rural**						
Rural	1.00			1.00		
Urban	1.42	1.07–1.87	0.014	1.01	0.65–1.58	0.955
**Region**						
Sughd	1.00			1.00		
Dushanbe	0.48	0.30–0.77	0.002	1.24	0.69–2.23	0.467
Khatlon	0.40	0.28–0.55	<0.001	1.19	0.72–1.96	0.504
DRS	0.51	0.36–0.74	<0.001	1.08	0.62–1.87	0.793
GBAO	0.35	0.23–0.54	<0.001	3.36	1.77–6.38	<0.001
**Firstborn**						
Yes	1.40	1.10–1.78	0.006	1.40	0.92–2.11	0.113
No	1.00			1.00		
**Mother’s decision-making**^d^						
Not involved	1.00			1.00		
Involved	1.26	0.99–1.60	0.060	0.63	0.41–0.98	0.043

Table shows association with migration when adjusted for all other variables in the table, with logistic regression analysis, without interaction terms. Based on weighted data (adjusted for clustering and stratification).

a Timely ANC: ANC started in the first trimester.

b p-values from Wald-test for association between covariable and timely ANC, resp. birthweight, adjusted for all other variables in the model.

c age is a continuous variable (months).

d mother’s decision-making: involved in decisions about health.

When adjusting for all potential confounders, there was very strong evidence that newborns whose fathers had worked abroad, had 37% lower odds to have LBW compared to newborns whose fathers had not worked abroad (adjusted OR 0.63, 95%-CI 0.44–0.89, p = 0.009). Adjusting for confounders made this relationship stronger (from crude OR 0.66). The father having higher education and mother being involved in decisions about health were the most significant confounders.

### 3.6 Other risk factors for timely ANC and low birthweight

The multivariable regression analysis in Table 4 also shows other factors than migration being associated with timely ANC and/or LBW when adjusted for all other variables.

Women over 30 years of age were less likely to start ANC on time (adjusted OR 0.67, 95%-CI 0.52–0.86, p = 0.002). Both maternal higher education (adjusted OR 1.77, 95%-CI 1.32–2.39, p < 0.001) and paternal higher education (adjusted OR 1.36, 95%-CI 1.07–1.73, p = 0.01) were associated with higher odds of timely ANC. Women in urban areas started ANC earlier (adjusted OR 1.42, 95%-CI 1.07–1.87, p = 0.01). Compared to the Sughd region, women in all other regions had 49–65% decreased odds of timely ANC. Women giving birth to their first child had 40% higher odds of timely ANC compared to those already having children (adjusted OR 1.40, 95%-CI 1.10–1.78, p = 0.006).

Regardless of migration status, and adjusted for all other variables, women whose husbands had higher education had 33% lower odds of having a child with LBW (OR 0.67, 95%-CI 0.45–1.00, p = 0.05). When women were involved in decisions about health, odds of LBW decreased with 37% (OR 0.63, 95%-CI 0.41–0.98, p = 0.04). There was no evidence for an association of LBW with maternal education levels. There was very strong evidence for a more than threefold increased odds of LBW in the mountainous GBAO region (when adjusted for migration and all other variables, OR 3.36, 95%-CI 1.77–6.38, p < 0.001), which could be related to the high altitude of GBAO (Yang et al., 2020). However, as this region only accounts for 1.7% of the study population, it had very little effect on the total outcomes.

The adjusted Wald test showed interaction in the association between migration and timely ANC for region (p < 0.0001), maternal age (p = 0.004) and maternal education (p = 0.003). Region was also an interaction factor in the association between migration and LBW (p < 0.0001). Wealth quintile was an important interaction factor for the relationship between timely ANC and migration (p < 0.0001), but not for the relationship of LBW and migration (p = 0.09). Because most of these variables are not binary, and because of restraints with survey data in Stata, separate ORs could not be obtained, and the final model is shown without interaction terms.

A sensitivity analysis for wealth quintile showed a mean VIF of 1.66, ranging from 1.08 to 3.59 (highest values for wealth quintiles 4 and 5). This suggests low levels of collinearity. Including wealth quintile in the logistic regression model had a small effect on timely ANC (adjusted OR 0.77, 95%-CI 0.63–0.95, p = 0.01) and no effect on LBW (adjusted OR 0.63, 95%-CI 0.44–0.90, p = 0.01). For urban women, the odds of timely ANC reduced to 1.25 and was no longer significant (95%-CI 0.91–1.73, p = 0.16).

## 4 Discussion

The aim of this study was to assess whether there is an association between fathers working abroad and the health and pregnancy outcomes of their left-behind wives and newborns in Tajikistan. The study found 20% lower odds for starting ANC on time for women with a migrant husband compared to women with a non-migrant husband. Children of migrant fathers were 37% less likely to have LBW compared to children of non-migrant fathers when adjusted for age, gender, maternal age, education of the mother and father, urban setting, region, firstborn child and mother’s decision-making position in the family.

Literature about the association of paternal migration with maternal and newborn health is scarce. Most studies on children’s health focus on internal (rural-to-urban) migration and were mostly conducted in China (Fellmeth et al., 2018; Račaitė et al., 2021).

LBW is a known risk factor for mortality and morbidity (Blencowe et al., 2019). Starting ANC in the first trimester benefits maternal and newborn health through interventions like dietary advice and checking the condition of mother and child (WHO, 2016). In an analysis based on the Tajikistan 2012 DHS, starting ANC in the first trimester was associated with a 300 *g* increase in birthweight compared to those starting later (Habibov et al., 2017). Migration was not taken into account here (SA, MOHSP and ICF, 2013). In contrary, our study found that wives of migrants started ANC later but their children had higher birthweights, although this is not necessarily occurring in the same groups.

A study in Sri Lanka also found an increase in birthweight associated with male migration and attributed this to increased use of private healthcare and improved nutrition during pregnancy resulting from remittances (Jayatissa et al., 2022). Private healthcare is very limited in Tajikistan, and most healthcare is paid out of pocket, even ANC (Khodjamurodov et al., 2016; Nabieva and Souares, 2019). There was no increase in the number of ANC visits for migrant wives in our study. One explanation for the effect of migration on birthweight in Tajikistan could be improved nutrition following the receival of remittances, although this could not be confirmed with our data. Male migration could also have led to women’s increased healthcare utilisation other than ANC visits, for example increased use of vitamins, medication and ultrasounds, and affected birthweight, but information about this is not available.

An association of male migration with a later start of ANC has not been described outside of Tajikistan. A study from northern Tajikistan suggested that the role of mothers-in-law became stronger after a husband’s migration and created barriers to ANC, as especially older mothers-in-law doubted the benefit of ANC (Nabieva and Souares, 2019). Due to the cross-sectional nature of our DHS data, any changes in decision-making power for women when husbands migrated or returned could not be investigated. Also, we found lower odds for timely ANC attendance among women over 30 years of age, but how much this is also influenced by having older mothers-in-law cannot be concluded from this study.

Other hypotheses for starting ANC late could be that a newly pregnant wife delays starting ANC till the husband has left, or financial reasons e.g. spending money on an airplane ticket rather than healthcare or waiting for the husband to send remittances. Besides this, some men restrict where their wives can go during their absence.

Other factors besides migration associated with timely ANC and higher birthweight in our study, were fathers with higher education and mothers involved in decision-making about health. Though maternal education is a known factor affecting newborn and child health, the role of paternal education has been studied much less, but associations with a reduction in child mortality and undernutrition have been described (Balaj et al., 2021; Vollmer et al., 2017). This means that fathers also matter for the health of their children. Parental education levels and mother’s involvement in decision-making about health show that women’s autonomy is important in health-seeking behaviour and health outcomes. A study based on the Tajikistan 2012 and 2017 DHS concluded that an increase in women’s autonomy improved maternal care utilisation, which echoes findings from other countries about the relationship between women’s autonomy and health (Auchynnikava and Habibov, 2021).

The relationship between migration and wealth quintile is complicated. Wealth can both be a driver for migration and change because of migration. Wealth can influence selection into migration, although this relationship is not linear. Those from the richest quintile were less likely to leave for migrant work as they have lower need for it. Those from the poorest quintile may not be able to afford to go abroad, or they have sold everything they have to migrate, but are not receiving remittances and the family is left in poverty. When wealth quintile is not only a reason for migration, but also a consequence of migration, it could play a causal role on the association between migration and health or healthcare utilisation. When wealth selects for migration, this could confound the effect on birthweight. We decided to not include wealth quintile in the logistic regression model for these reasons and because of possible collinearity with urban setting. Other studies have discussed these same issues but still included socioeconomic status in the regression model (Vollmer et al., 2017).

This study did not include psychosocial factors. It is not known whether Tajik women experience more stress or mental health problems when their husbands are away. Some studies suggested an increase in mental health problems due to husband’s migration (Aryal et al., 2019; Graham et al., 2015; Lu, 2012). How this plays out will depend on the local context, like family structure and available support systems (Edelblute and Altman, 2018). Many Tajik women live with their in-laws which could influence the experienced support and stress when a husband migrates. There are high rates of domestic violence in Tajikistan, and besides the husband the mother-in-law is also noted as a perpetrator (Haarr, 2007). Tajik women may experience either increased or reduced stress when their husband migrates.

The role of the husbands needs to be considered as well. A survey in Tajikistan revealed that men are interested to learn more about topics related to pregnancy and health (USAID, 2021). Future studies could look at how men could be involved more, how they could support a (pregnant) wife well from distance, and how women’s autonomy can be improved.

### 4.1 Strength and limitations

This is the first time, as far as is known, that DHS data was used to investigate an association between husband’s migration, maternal health-related behaviours and newborn health outcomes. One advantage of using DHS data is the large study sample.

Because of the cross-sectional nature of the survey, no causal relationships could be investigated. There was no data about when and for how long the husband was away, other than ‘a minimum of three months in the last three years’. In practice, men often stay away longer (IOM Dushanbe, 2009). It was also unknown whether the husband was away before, during or after the pregnancy. This could have caused bias in the association between husband’s migrant status and maternal and newborn health and the real associations may be stronger or weaker than found here.

Confounders may have been missed, leaving residual confounding. Selection of main outcomes based on crude associations, though justified by the primarily exploratory nature of the study, could cause bias. The use of weightings could have introduced additional bias to the study if some factors were overrepresented in groups that had fewer women sampled into the study. The data was self-reported and could have introduced recall bias. Although grouping the responses about mothers’ decision-making in ‘involved’ (alone or with husband or others) or ‘not involved’ is common practice and used in many studies, including those from DHS themselves, one could argue that this assumption misclassifies autonomy.

Birthweight is an outcome that can especially ‘suffer’ from issues with recalling, measuring errors or heaping at certain values (Blencowe et al., 2019). In Tajikistan there is a tendency to weigh newborns with clothes on (personal communication with midwives and mothers), as Blencowe also described. Missing data for birthweight was found in the literature to be associated with lower education levels and higher birth order (Habibov et al., 2017). Though birthweight was only missing for 8%, we found a weak association for missing birthweight with migration status and a stronger association with many of the covariates in the current study.

### 4.2 Conclusion

This study shows that there is an association between a husband being away for labour migration or not leaving the country, and the health-related behaviours of their wives and health outcomes of their newborns. Women started ANC later but their babies had higher birthweights when a husband had migrated. It is therefore impossible to conclude that labour migration is ‘good’ or ‘bad’ for those left behind. The situation is much more complex, and this association is influenced by the region they live in, by fathers’ education levels and mothers’ involvement in decisions about health.

The results found in this study cannot be generalised to other settings, as many factors are specific for Tajikistan, including high migration rates, a largely absent private care sector, and a family structure where most women live with in-laws. A comparison of the current study with a future Tajikistan DHS would be good, to investigate whether the associations remain the same.

More research is needed to understand whether there is a causal relationship and what the mechanisms for this association could be, such as the role of remittances and the duration and timing of migration. Future studies also need to cover psychosocial factors, including experiences of domestic violence and available support.

Based on our findings and previous literature, we hypothesize that improving both father’s support to a pregnant wife, even from distance, and increasing women’s autonomy could improve ANC uptake and birthweight in Tajikistan. To accomplish this, policy makers could stimulate all women to start ANC in the first trimester, addressing the husbands and mothers-in-law about the importance of ANC before husbands leave the country. Also, drawing on the literature, we suggest that creating more jobs for men and improving access to healthy nutrition for all pregnant women might reduce LBW and improve the health of the future generation.

## CRediT authorship contribution statement

**J․Mirjam Oudijk:** Writing – review & editing, Writing – original draft, Visualization, Validation, Project administration, Methodology, Formal analysis, Data curation, Conceptualization. **Jelle Stekelenburg:** Writing – review & editing, Writing – original draft, Supervision.

## Declaration of competing interest

The authors declare that they have no known competing financial interests or personal relationships that could have appeared to influence the work reported in this paper.
